# Vinyl Chloride and U.S. EPA Research

**DOI:** 10.1289/ehp.113-a653

**Published:** 2005-10

**Authors:** Courtney M. Price

**Affiliations:** CHEMSTAR, American Chemistry Council, Arlington, Virginia, E-mail: courtney_price@americanchemistry.com

A commentary by Sass et al. (2005), “Vinyl Chloride: A Case Study of Data Suppression and Misrepresentation,” is itself a case study in misrepresentation. The inclusion of such an article in this peer-reviewed publication stands in contrast to its stated mission to publish “balanced” and “objective” information. Sass et al. (2005) did not include or address recent studies in characterizing the weight of the scientific evidence related to vinyl chloride and made inaccurate and unsupported allegations about the integrity of U.S. Environmental Protection Agency (EPA) scientists and the rigorous peer review process utilized by the U.S. EPA.

Sass et al. (2005) asserted that there is a “scientific consensus that [vinyl chloride] is a multisite carcinogen in humans and experimental animals,” referencing 21 articles, only 3 of which were published during the past 15 years. They failed to mention or seriously discuss 7 articles noted below that were published in scientific journals since 1997 and update many of the studies Sass et al. cited and reach the opposite conclusion. These and other recent peer-reviewed studies and reviews fully support the U.S. EPA’s conclusion that “the association [between vinyl chloride and cancers other than the liver] is weak and any estimated increase in mortality from cancer at these sites is likely to be less than for liver cancer” (U.S. EPA 2000).

Authors of these articles include Aaron Blair, the chief of the Occupational Studies Section of the National Cancer Institute (NCI), who stated that epidemiologic evidence shows a strong exposure–response relationship for angiosarcoma of the liver, but not for other types of cancer (Blair and Kazerouni 1997). In a more recent review, McLaughlin and Lipworth (1999) reached the same conclusion:

Occupational vinyl chloride exposure has not been conclusively causally linked to any adverse health outcome, with the exception of angiosarcoma of the liver.

Even more recently, Bosetti et al. (2003) stated that

The aggregate data are reassuring in excluding any excess risk of death from lung, laryngeal, soft tissue sarcoma, brain and lymphoid neoplasms, as well as cirrhosis.

Recently published updates of cancer incidence in European and American industry-wide cohorts of workers exposed to vinyl chloride provide a firm basis for the conclusion that vinyl chloride exposure is not causally associated with brain cancer and the other tumors mentioned by Sass et al. (2005). The European study (Ward et al. 2001) was conducted by scientists affiliated with the National Institute of Occupational Safety and Health (NIOSH) and the International Agency for Research on Cancer (IARC). The authors found no evidence of an increase in cancers other than the liver. Similar, though less definitive, results were published by Mundt et al. (2000) in an update of the American cohort. A recent meta-analysis of these cohorts by IARC scientists further supports the conclusion reached by the U.S. EPA (Boffetta et al. 2003).

Given the strength and uniformity of the evidence supporting the U.S. EPA’s position, it is striking that Sass et al. (2005) did not address it. Instead, they claimed that the U.S. EPA yielded to advocacy by chemical manufacturers, implying that the U.S. EPA relied in part upon unpublished data. As noted above, however, the articles upon which the U.S. EPA placed primary reliance are published, in a few cases, by academic scientists sponsored by industry (e.g., Mundt et al. 2000), but for the most part by scientists affiliated with some of the most prestigious government-supported organizations engaged in cancer research (e.g., NCI, IARC, NIOSH).

Finally, it is not accurate that industry unduly influenced the review process for vinyl chloride nor that the potency factors published in the IRIS (Integrated Risk Information System) database (U.S. EPA 2000) are insufficiently protective (Norman 2002). The former comment disparages the U.S. EPA scientists who spent 5 years and went through two external peer reviews to make sure that relevant current science was reflected. The latter fails to recognize that a pharmacokinetic (PK) approach to risk assessment was supported over 20 years ago by the National Academy of Sciences and that the PK model for vinyl chloride used by the U.S. EPA—which predicted the actual incidence of angiosarcoma of the liver in the early cohorts of exposed workers—has been peer-reviewed, published, and validated (Clewell et al. 2001; Reitz et al. 1996).
